# Cases, Illustrating the Efficacy of Paracentesis, in Abscesses of the Pleura

**Published:** 1829-03

**Authors:** 


ART. II.—*Cases illustrating the efficacy of Paracentesis, in abscesses of the Pleura.*

1. *A case of Empyema successfully treated by Paracentesis Thoracis.* by JEREMIAH H. BROWER, M. D.

J. Bolander, a stout, robust German, was attacked about the first of Nov. ult. after unusual exposure to the weather, with severe pain in the left side, attended with difficulty and pain in breathing, and dry, harrassing cough: he took some aperient medicine, was bled and sweated, which relieved the violence of the pain in some degree, the cough still continuing with occasionalfever. This state continued for three weeks, the patient still at times pursuing his occupation as a shoemaker; when he applied to me for advice, he complained of a dull obtuse pain in the left side; inability to lie upon the right side, an attempt at which produced an immediate sense of suffocation and faintness; the pain but little increased by full inspiration; countenance pallid, and emaciation great and progressive; he coughed incessantly, expectorating in large quantities, fœtid purulent matter; the offensiveness of which produced constant nausea and vomiting: regular hectic paroxysms were established. About two weeks from the commencement of his illness, he experienced a severe rigor, which lasted for half an hour, a few days after which, his fever assumed the form of regular hectic exascerbations. his pulse was small and frequent; bowels regular, & tongue clean. Upon attentive examination, the left side of the chest was perceptibly larger than the right; by percussion of the left side, in the lower portion, a dull, dead sound as of a solid body was elicited, while in the upper portion, above the fourth rib, and in the axillary region, it was unusually clear and resonant; the right side yielded a hollow sound when struck. By applying a cylinder to the left side, not the least respiratory murmer could be perceived in the lower portion of the chest; in the upper portion, and under the axilla it was indistinct, and a peculiar hissing sound was perceptible, which I can compare to nothing so aptly, as displacing water in a vessel by blowing through a tube. In the right side, throughout its whole extent, a distinct respiratory murmur was perceived, and on that side the action and rythm of the heart were much more distinct than on the left; the patient remarked, that for several days past, he was sensible of the splashing of a fluid in the left side, and upon succussion, a fluctuation was distinctly ascertained. Under these circumstances there could be no doubt of a purulent effusion, into the left cavity of the chest having taken place from an abscess in the cellular structure of the lung, into which one or more of the ramifications of the bronchiæ opened, and that an operation for its evacuation, presented the only prospect of relief or recovery. This the patient anxiously desired, and accordingly, on the first of December, I proceeded to it by an incision between the sixth and seventh ribs at their angles, cautiously avoiding the intercostal artery, and upon laying bare the pleura costalis a small sized trocar was pushed through it. A rush of fœtid gas only, immediately followed it; the patient had some years since, suffered from a protracted pleuritic affection of the same side, I thought it probable that extensive adhesions had supervened, and now obstructed the exit of the matter contained in the sac of the pleura. As the patient was considerably exhausted, I left the canula in for an hour, and then withdrawing it, passed in and downwards a large gum elastic catheter, by which three pints of thick fœtid pus were slowly evacuated; the patient could now, by taking a full inspiration and closing the larynx, expel the air from the wound in the parietes of the chest. Great and immediate relief from the cough, dyspnœa and pain was obtained. A bougie was left in the opening for forty-eight hours, when, the cough returning, the catheter was again introduced and nearly two pints of bloody purulent matter discharged. The cough &c. abated, and my patient began to improve rapidly in appearance; his appetite was good, and sleep regular and refreshing. He continued to do well until the fifth day after the operation, when in a violent fit of coughing the tent which was kept in the wound, suddenly burst forth and was followed by a discharge of a pint and a half of the same sanguineo-purulent fluid as before. From this period his health and strength rapidly and steadily improved, and in four weeks from the operation, he was able to assume his work at the bench as usual; no inflammatory or untoward symptoms followed. The medical treatment was confined to mild asperients, with an occasional dose of Dover's powder at night, and towards the close, tonics and generous diet: the wound became fistulous, but by a few applications of argenti-nitras, it healed kindly. The left side now yields a sound clearer than the right, and is evidently less prominent than before the operation.

I have been induced to report this interesting case, from an impression that we are too much disposed to abandon, as hopeless, prolonged diseases of the cavity of the chest, from a fear, (perhaps in many instances groundless,) that the pulmonary structure has suffered so much as to preclude the possibility of relief; but in the present advanced state of our knowledge of the diseases of the chest, the result of extensive and persevering pathological research, and the increased certainty in their diagnosis, resulting from the attentive employment of the stethoscope and percussion, may we not indulge the hope, that many of the cases, heretofore abandoned as unsusceptible of even partial or temporary relief, may, by a more energetic and discriminating practice, be brought to a happy termination. The result of the above case, seems to justify the following conclusions, viz: That when it is ascertained, that a sero-purulent effusion into the cavity of the chest exists, unconnected with disorganization of the cellular tissue of the lungs, the operation of paracentisis thoracis, speedily gives relief, and often effects a cure; and that even when such structural disorganization is in progress, may be the means of relieving the sufferings of the patient, and prolonging life; and that we can be greatly assisted by auscultation and percussion, together with measurement of the two sides of the chest, in cases of empyema, in determining the presence of a fluid, and the best spot for the incision, and that in such cases, even under the most gloomy appearances, we should not despond, as recovery partial or total, is possible under the most unfavorable circumstances.

2. *An account of a case Empyema, consequent upon Measles, cured by Paracentesis.* By the Editor.

Measles, of a type decidedly phlogistic, prevailed in Cincinnati throughout the spring of the year 1813. Among its subjects, was Matilda Harsher, aged 9 years, a child well developed and previously in good health. Instead of the antiphlogistic treatment which the epidemic demanded, her physician relied chiefly on Bateman's drops. Under a plan so stimulating, a cough and fever, as might have been expected, continued still to harrass her after the subsidence of the eruption.

Several weeks subsequent to this event, about the first of June, I was requested for the first time to visit her. I found her labouring under fever, the paroxysms of which were followed by colliquative sweats. Her pulse beat upwards of 150 in a minute, with great irregularity and feebleness. Her cough was dry; respiration difficult, and sense of suffocation distressing. She was unable to lie in a recumbent position, or on her right—the sound side. Her emaciation was decided. All the symptoms, in short, were discouraging.

On examining her chest, I found it out of shape. The upper part of the left side was manifestly enlarged and sore to the touch, especially below the nipple and in another spot over the false ribs. The cellular tissue was not œdematous. The heart could not be felt on the left side; but was found palpitating with great frequency and irregularity in the right side, at a point nearly corresponding with its usual position in the left. The right side, thus compelled to afford a lodgement for that organ, had lost *its* proper form. The ribs had bulged out, so as to present a rounded and irregular tumour, in the region of the right breast. As the important discoveries in auscultation, with which Lænnec has favored the profession, had not then been made, the stethoscope of course, was not tried.

The alarming situation of my little patient, required that something should be promptly done, and I administered an emetic and applied a blister to the left side; but these prescriptions, for which no adequate reason could, perhaps, be assigned, afforded not the least relief; and, in three days, her paroxysms of dyspnœa, and her inability to lie down had greatly increased.

An operation now seemed to be indispensable. On re-examining the affected side, the 5th, 6th, and 7th ribs appeared separated from each other, and there was a manifest fluctuation within. Between the 5th and 6th ribs a soft swelling, not very prominent, was obvious; and this was the spot selected for the puncture.

A horizontal incision, an inch and a half in length, was made through the skin and cellular tissue; when, instead of meeting with the intercostal muscle, the knife passed immediately through the pleura. The flesh at this spot had in fact been absorbed. A pint of 'laudable pus' was forthwith discharged. The wound was then dressed with lint and a poultice; but it continued to discharge copiously through the following night, and more sparingly the next day. The second night it ceased, and on the morning of the third day, the cut edges of the pleura seemed to have united. They were forced asunder, and a leaden canula introduced and left in the wound. By this measure, the evacuation was re-produced and continued with some copiousness, till about the fifth day after the operation, by which time we estimated, that she had lost at least three quarts. Thenceforth it was moderate.

Great and almost instantaneous relief resulted from the operation:—The chest diminished in size and deformity; the pulsations of the heart in the right side, became less violent, and soon fell to 110 or 12 in a minute, with great improvement in their rhythm; her cough moderated, and she could lie with her head and shoulders much lower.

She was put forthwith on the use of the Bark, and continued it for twenty months, during which she took thirty-two ounces. About a year after the operation, when I was no longer in attendance upon her, she was seized, as I have since understood, with a severe ague and fever. It might be supposed to have been hectic, but that the shake was protracted and violent, and the type of the disease changed, after a time, from quotidian to tertian, At the onset of this new malady and throughout its whole course, she was still using the Bark.

The canula was worn for twenty-eight months. In the latter part of this long period, she had but little cough or discharge, and was able to go to school. After it was withdrawn she had fits of general weakness, nausea, and sense of sinking about the chest; but within a few weeks these symptoms left her, and she ever since enjoyed good health.

After many years of separation from my patient, we lately had several professional interviews, and this evening (March 23, 1829,) she permitted me to examine her thorax.

The heart has returned to its proper situation, and can no longer be felt in the right side. By the stethoscope I ascertained, that its size and contractions are natural. The chest generally sounds tolerably well; but is much more sonorous just below the spot where the incision was made, than at any other point, indeed more so than is common. Neither at that spot, nor any other to which the cylinder was applied, can pectoriloquism be perceived. The respiratory murmur is no where loud. It can indeed, be scarcely heard, except in the superior parts of the chest; but is at least, as perceptible in the side affected as the other. Pressure gives her no pain or dyspnœa, except when made on the spot that was punctured; which is still tender, and although 13 years have elapsed since the orifice closed up, the cicatrix is still red. Measured from the ensiform cartilage to the spine, the affected side is about half an inch smaller than the other.

She lies equally well on her back or either side, and so far from requiring her head and shoulders to be raised, she prefers them unusually low; she can ascend a stair case or a hill, without any difficulty of breathing; she has no cough; finally, when at any time she has experienced a pain in the side, it has been in the one opposite to that affected with the abscess. She is now married and has one child.

Remarks.—This case in its origin was a striking example of the pernicious effects of a stimulating treatment in measles. If the operation had not been performed, the abscess *might*

have broken externally, but before the pleura and skin could have been absorbed, she would probably have died; the operation, therefore, may be said to have saved her life. The occurrence of ague and fever while the Bark was taken daily, was certainly not to have been expected. The canula was worn much longer than was necessary. We learn from it that the cavity of the pleura will bear, with impunity, a long continued exposure to the atmosphere.

---

3. *Notes of a case of Empyema from chronic pleurisy, in which the operation gave relief.* By the Editor.

William Davis, aged 35 years, residing in or near Louisville, was attacked with ague and fever in June 1826, and had occasional relapses for nine months. For three months more was pretty well; when he was seized with a pain in his right hip which yielded, as he supposed, to the shower bath. Soon afterwards—in September—he was seized with pleurisy in the right side, for which, under the care of Dr. Galt and Dr. Dalton, he was bled four times and blistered many more.—Considerably relieved, but still labouring under pain, cough, and dyspnœa, he came, in October, to the neighbourhood of Cincinnati, and called in the aid of a 'Dutch doctor;' who attended him until he became my patient in February. During that period, he was bled once and blistered several times. Of the medicines ordered for him I could learn nothing, except that one of the mixtures contained the oil of peppermint, and greatly aggravated his symptoms whenever he took it.

When he came to the city and put himself under my care, his prominent symptoms, were an incessant cough with copious expectoration; a pulse beating from 120 to 140 in a minute, weak and elastic; irregular hectical exascerbations; copious night sweats; an inability to lie in a recumbent posture; insomnolency; dyspnœa, greatly increased by the least exertion; œdema of the lower extremeties, which had existed all winter; occasional diarrhœa; sallow complexion; pain in the right side of the chest and abdomen; debility so great as scarcely to be able to walk across his chamber; and general emaciation.

External examination of the chest.—*Form and size.* To the eye, directed towards the face of the patient, his left side appeared more convex than the right, which in the lower part seemed rather flattened; but below, and not far from the right breast, there was a considerable bulging out of the ribs, and thickening of the superincumbent soft parts, which were œdematous. The most prominent point of this tumour was red. A fluctuation was perceptible. Measured over the swelling the right side was the larger; but above it the dimensions were reversed.

*Pressure.*—He bore pressure well over the whole chest, except on the tumour, where it hurt him. He also felt pain in the same part on coughing.

*Percussion.*—On being struck, the whole of the left side was sonorous; but the right was inelastic and emitted no hollow sound, except in the region of the spine, high up.

*Auscultation.*—In using the stethoscope, the respiratory murmur was audible over every part of the left side; but could only be perceived in the superior part of the right. Near the spine on this side, there was decided pectoriloquism; which was, also, perceptible in a slighter degree on the left.

*Heart.*—The rhythm, impulse, and sound of this organ, were natural. In the *right* side, especially in the axilla, the sound was louder than in the left. Posterior to the tumour, there was a metallic tinkling connected with the contraction of the ventricles, which could be heard in no other part of the chest.

From the general symptoms my first impression, of course, was, that the patient laboured under an incurable dyspeptic consumption; but after an examination of the chest, and the discovery of a purulent accumulation in the side, the case assumed to my mind the character of chronic pleurisy combined with hepatic derangements, the offspring of intermittent fever.

Under this state of things, the operation for empyema seemed to offer the only hope of relief which remained for the unfortunate patient; and from it, but temporary benefit could reasonably be expected.

On the 10th of February I performed the operation, which was nothing more than the introduction of a broad lancet, as in opening a common abscess. It evidently entered a cavity, but no pus followed its withdrawal. The patient, however, was directed to bend his body forward, when a free discharge of purulent matter, to the quantity of 6 or 8 oz. ensued, The canula was introduced, and cotton applied over it to absorb the pus, which for many days continued to flow copiously. The tube was not worn long, as it seemed unnecessary; but the discharge continued, gradually decreasing, till the patient set off for the city of New York, in the month of June last; and it appears, by the accounts received from him, that it still remains. Immediately after the operation, he was put on the use of the Bark, opium, the blue pill, and prepared chalk, the last of which was required to moderate his diarrhœa. He also, at different times, took diuretics for the relief of his dropsical symptoms.

It remains to speak of the effects of the operation. These were, in all respects, as salutary as could have been expected. His cough, expectoration and dyspnœa were signally diminished; his pulse experienced a great abatement in its frequency; he slept much better, and could lie with his head and shoulders, at the usual elevation; his night sweats recurred but seldom; his strength returned, so that he was soon able to walk about the city, and ascend to its most elevated places; his digestive functions were ameliorated, and he recovered a great deal of flesh. The œdema of his lower extremities, however, continued, though it did not increase; the diarrhœa was still prone to return; and, about three months after the operation, he suffered an attack of violent pain in his knee and ankle joints.

Before his departure for New York, I examined his thorax. The symmetry of its two sides was nearly restored; but the respiratory murmur had not yet become audible in the parts where it was suspended; nor had its sonorousness returned.

This was, evidently, not a simple case of chronic pleurisy, occasioning sero-purulent effusion. The dropsical infiltration into his lower extremities, and the pains of his joints, his irregular bowels, pain in the region of the liver, and sallow complexion, sufficient to indicate a hepatic disease; while his copious expectoration before the operation, and the inaudible and inelastic state of the right side render it extremely probable, that the right lobe of the lungs is hepatized, or otherwise deranged in its structure.

In the midst of so much complication, all that could be reasonably expected, was the relief that would follow the evacuation of the pus accumulated in the cavity of the pleurisy. The amount of this palliation has been stated; and must, I think, be acknowledged to have been sufficient to justify the operation, and recommend its adoption in all cases of a similar kind.

